# Toll-Like Receptor 4 Promoter Polymorphisms: Common *TLR4* Variants May Protect against Severe Urinary Tract Infection

**DOI:** 10.1371/journal.pone.0010734

**Published:** 2010-05-20

**Authors:** Bryndís Ragnarsdóttir, Klas Jönsson, Alexander Urbano, Jenny Grönberg-Hernandez, Nataliya Lutay, Martti Tammi, Mattias Gustafsson, Ann-Charlotte Lundstedt, Irene Leijonhufvud, Diana Karpman, Björn Wullt, Lennart Truedsson, Ulf Jodal, Björn Andersson, Catharina Svanborg

**Affiliations:** 1 Department of Microbiology, Immunology and Glycobiology, Institute of Laboratory Medicine Lund, Lund University, Lund, Sweden; 2 Singapore Immunology Network (SIgN), Biomedical Sciences Institutes, Agency for Science, Technology, and Research (A*STAR), Singapore, Singapore; 3 Department of Urology, Institute for Clinical Sciences Lund, Lund University Hospital, Lund, Sweden; 4 Department of Biological Sciences, National University of Singapore (NUS), Singapore, Singapore; 5 Department of Pediatrics, Institute of Clinical Sciences Lund, Lund University, Lund, Sweden; 6 Department of Pediatrics, The Queen Silvia Children's Hospital, Gothenburg University, Gothenburg, Sweden; 7 Department of Cell and Molecular Biology, Karolinska Institutet, Stockholm, Sweden; Instituto Gulbenkian de Ciência, Portugal

## Abstract

**Background:**

Polymorphisms affecting Toll-like receptor (TLR) structure appear to be rare, as would be expected due to their essential coordinator role in innate immunity. Here, we assess variation in TLR4 expression, rather than structure, as a mechanism to diversify innate immune responses.

**Methodology/Principal Findings:**

We sequenced the *TLR4* promoter (4,3 kb) in Swedish blood donors. Since TLR4 plays a vital role in susceptibility to urinary tract infection (UTI), promoter sequences were obtained from children with mild or severe disease. We performed a case-control study of pediatric patients with asymptomatic bacteriuria (ABU) or those prone to recurrent acute pyelonephritis (APN). Promoter activity of the single SNPs or multiple allelic changes corresponding to the genotype patterns (GPs) was tested. We then conducted a replication study in an independent cohort of adult patients with a history of childhood APN. Last, *in vivo* effects of the different GPs were examined after therapeutic intravesical inoculation of 19 patients with *Escherichia coli* 83972. We identified in total eight *TLR4* promoter sequence variants in the Swedish control population, forming 19 haplotypes and 29 genotype patterns, some with effects on promoter activity. Compared to symptomatic patients and healthy controls, ABU patients had fewer genotype patterns, and their promoter sequence variants reduced TLR4 expression in response to infection. The ABU associated GPs also reduced innate immune responses in patients who were subjected to therapeutic urinary *E. coli* tract inoculation.

**Conclusions:**

The results suggest that genetic variation in the *TLR4* promoter may be an essential, largely overlooked mechanism to influence TLR4 expression and UTI susceptibility.

## Introduction

The innate immune response is essential for survival during early stages of infection. Toll-like receptors (TLRs) are critical sensors of microbial attack and effectors of the TLR dependent innate defense enable the host to eliminate pathogens that otherwise would cause disease or mortality [1], [2], [3]. The TLR structure is relatively well conserved throughout evolution, consistent with the involvement of specific TLR domains in multiple, specific ligand interactions [4], [5], [6] and the recruitment to the Toll/interleukin-1 receptor (TIR) signaling domain of adaptor proteins, which are essential for TLR-signaling [5], [7], [8], [9], [10], [11]. Given their crucial role as sentinels of the innate immune defense, TLR structure and function are tightly regulated [12]. Numerous attempts have been made to identify variation affecting TLR structural genes and resulting disease susceptibility, but structural gene polymorphisms are relatively rare [13] and their contribution to human disease remains unclear.

UTIs are among the most common bacterial infections in man, and remain a major cause of morbidity and mortality [14]. A subset of disease-prone individuals is at risk for recurrent acute pyelonephritis (APN), severe renal dysfunction and even end-stage renal disease and genetic markers of susceptibility have recently been described in this patient group [15], [16], [17]. Asymptomatic bacteriuria (ABU) is a more common condition than APN in most age groups. Despite high bacterial numbers in urine >10^5^ cfu/ml, the patients do not develop symptoms and innate immune responses to infection are low [18]. A molecular/genetic basis for the low immune response in this patient group has not been identified, however. In the murine UTI model, Tlr4 controls the innate immune response to *Escherichia coli* and *Tlr4* ^-/-^ mice develop ABU rather than severe infection [19], [20], [21] suggesting that reduced mucosal Tlr4 function may protect the host against symptomatic infection. Low TLR4 expression levels were also observed in children with ABU [22], thus supporting the relevance to human disease of the observations in the murine model.

In this study, we examined whether differences in TLR4 expression levels may result from *TLR4* promoter sequence variation and if diversity of innate immune responses could be attributed to variant TLR4 expression. The results indicate that TLR4 expression levels influence the innate immunity response and potentially link *TLR4* promoter sequence variation to human disease susceptibility.

## Methods

### Patients and controls

To investigate if *TLR4* promoter variation might influence UTI susceptibility, we enrolled two highly selected, UTI prone study populations with a long history of either acute pyelonephritis (APN) or asymptomatic bacteriuria (ABU) in childhood (The clinical characteristics of patients and controls included in this study are provided in supplementary table 1 (Table S1)). The patients in study 1 were selected from all children with UTI, who were being referred to one pediatric nephrologist (DK) and followed for at least six years at the Department of Pediatrics, Lund University Hospital. The diagnosis of APN was based on a febrile infection (≥38.5°C) with significant bacteriuria, C-reactive protein (CRP) >20 mg/l and lack of symptoms of other infections. A diagnosis of asymptomatic bacteriuria was based on at least three consecutive urine cultures yielding the same bacterial strain (>10^5^ cfu/ml of urine) in a child with no symptoms of UTI and no CRP increase. Patients with no evidence of symptomatic UTI were assigned to the primary ABU group (7 boys and 9 girls, median age 5, range <1–20). Children who developed ABU after having completed antibacterial therapy for a prior symptomatic infection but with no further symptomatic episodes were assigned to the secondary ABU group (4 boys and 10 girls, median age 7, range 1–13). The APN group comprised 21 children (3 boys and 18 girls, median age 8.5 years at blood sampling, range <1–17 years) with a history of APN, who had not developed ABU at any time during follow-up.

Study 2 included 62 adult white patients with a history of childhood APN, who participated in a study of febrile UTI in the 1970ies and were reinvestigated to evaluate genetic associations between UTI morbidity and long-term effects of these infections on health and kidney function, about 30 years after the initial UTI episode [23]. Patients in this group, who consistently developed APN, were assigned to the APN group, while patients who developed ABU secondary to an APN episode were assigned to the secondary ABU group.

Pediatric age-matched controls (n = 39) were enrolled at the pediatric outpatient clinic or when admitted for elective surgery for diagnoses unrelated to infection. They had negative urine cultures at the time of sampling and no recorded history of UTI. The controls were white and the families were not consanguineous. Adult healthy blood donors (n = 200) from southern Sweden were included to assess *TLR4* sequence variation in the population.

Informed written consent was obtained from all participants or their parents/guardians. The study was approved by the Ethics Committee of the medical faculty, Lund University, Sweden (LU106-02, LU236-99).

### Human therapeutic inoculation protocol

The prototypic ABU *Escherichia coli* strain 83972 has been extensively used for therapeutic urinary bladder colonization in patients with dysfunctional voiding and chronic UTI [18], [24], [25], [26], [27]. Briefly, the patient is treated with appropriate antibiotics if needed to eliminate pre-existing bacteriuria. After an antibiotic free interval, the patient is catheterized, 30 ml of *E. coli* 83972 (10^5^ cfu/ml) is instilled, and the catheter is removed. The patients in this study (n = 15) had neurogenic bladder disorders and a history of recurrent UTI (3–4 UTI/year) during the last two years but no APN episodes. Bacterial inoculations were performed at the Urology Out-Patient Department, Lund University Hospital under rigorous control and no adverse effects were observed. Urine samples were obtained prior to and at standardized times after inoculation, for bacterial culture, verification of *E. coli* 83972 bacteriuria and for quantification of neutrophil numbers with Bürker chamber and Interleukin-6 (IL-6) and IL-8 levels by Immulite.

### Genomic DNA sampling

Genomic DNA was extracted from heparinised peripheral blood using QIAamp DNA Blood Midi kit. Primers were designed according to the published sequence for the *TLR4* promoter, starting 4.3 kb upstream of the ATG start codon (GenBank accession number: AF177765). Genomic DNA was PCR amplified and purified with a QIAquick PCR purification kit (Qiagen, Hilden, Germany) before being sequenced in both directions using nested primers (All primers are listed in Table S2. Patient sequences were base called and multi-aligned along with control sequences using PolyPhred and Phrap [28], visualized and manually compared using Consed [29]. Sequencing was done on a MegaBACE 1000 using a DYEnamic™ ET Dye Terminator kit (Megabace™) (Amersham Pharmacia Biotech).

### Pyrosequencing

The *TLR4* promoter variants detected by sequencing were confirmed on a Pyrosequencer PSQ 96 using a PSQ 96 SNP Reagent kit (Qiagen) and their frequency in the different patient groups was determined. The pyrosequencing primers were designed as shown in table S2 and used according to the manufacturer's instructions (Biotage).

### Genotype vector constructs

A 4.3-kb genomic DNA fragment comprising the human *TLR4* promoter was amplified from one patient with genotype pattern GPIV, the predominant GP in the Swedish population, using the Fermentas High Fidelity PCR system (29). In the heterozygote positions, the clone carried the minor allele as confirmed by sequencing. The product was cleaned with QIAquick PCR purification kit (Qiagen, Hilden, Germany), *Nco*I-cleaved and ligated into a *Nco*I-cleaved SAP-dephosphorylated pGL3basic vector. To create single mutations or multiple allelic variants, QuickChange XL site-directed mutagenesis kit (Stratagene) was used (The primers and cloning scheme for the different constructs is described in table S3).

### Transient transfections and dual luciferase reporter system assay

A498 renal carcinoma cells were cultured in 6-well plates in RPMI 1640 medium (5% FCS) at a density of 5×10^5^ cells per well. The cells were transiently transfected at 80% confluency using Fugene HD with the wild-type or mutant h*TLR4* promoter constructs (4:2), as well as an internal renilla luciferase control plasmid (pRL-TK) (Promega). Light emissions of firefly and renilla luciferase were measured using the Dual Luciferase Reporter System Assay (Promega). The firefly luciferase data was normalized against the Renilla luciferase.

To examine the response of the *TLR4* promoters to infection, the A498 human kidney cell line was exposed for 4 hrs to 10^8^ cfu/ml of the prototype uropathogenic *E. coli* strain CFT073, re-suspended in PBS after overnight growth on LB agar.

### Bioinformatics analysis of the TLR4 promoter

The human *TLR4* promoter sequence was compared pair-wise to the following sequences using BLAST [30]:

1. *Pan troglodytes* chromosome 9, reference assembly NC_006476.2.
2. *Bos taurus* chromosome 8, reference assembly NC_007306.3.
3. *Sus scrofa* chromosome 1, reference assembly NC_010443.1.
4. *Mus musculus* chromosome 4, reference assembly NC_000070.5.
5. *Rattus norvegicus* chromosome 5, reference assembly NC_005104.2.

**Figure 1:**
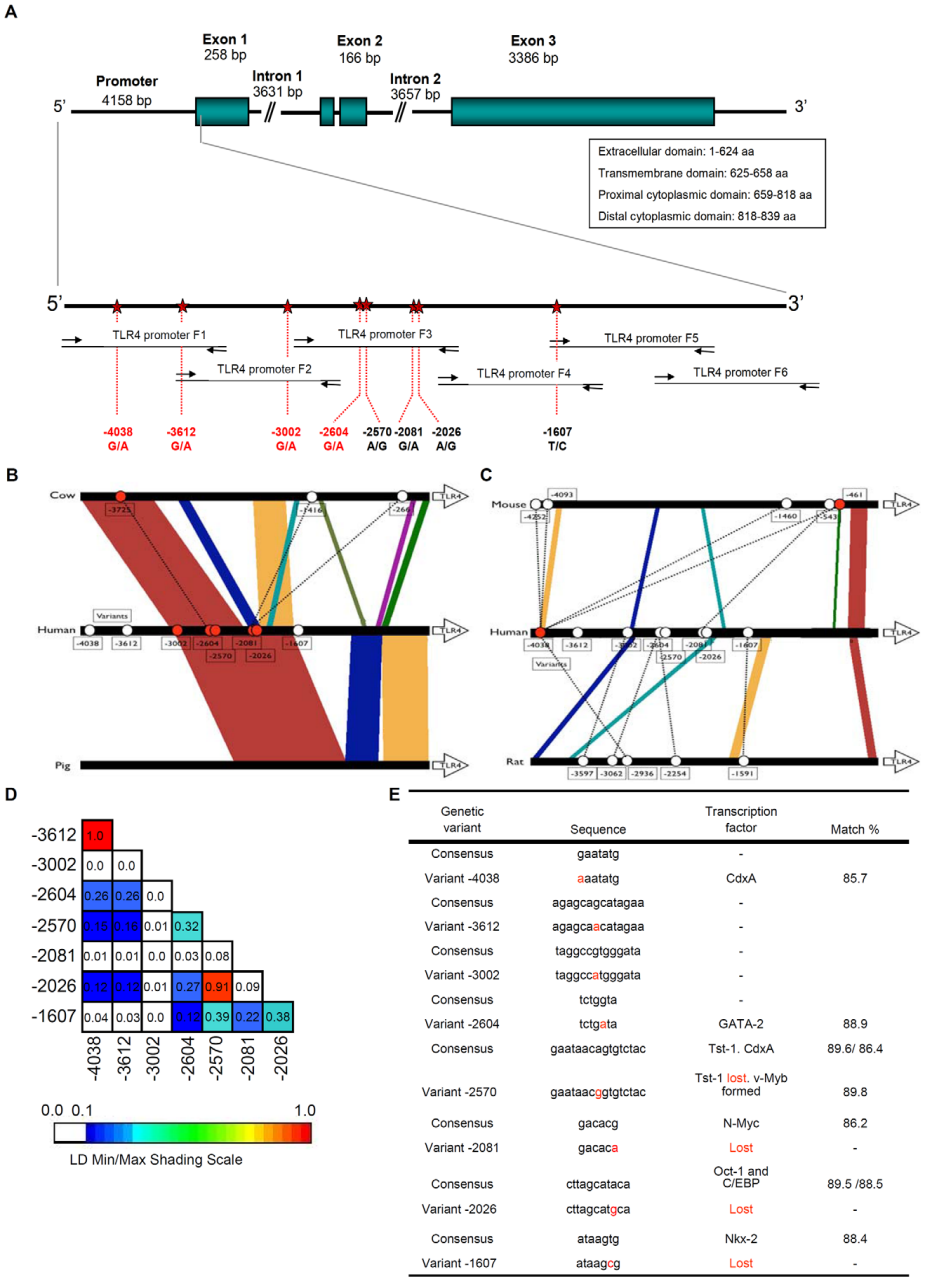
Polymorphisms in the promoter region of the *TLR4* gene. A. Eight sequence variants were identified by direct sequencing of PCR products from the distal promoter region of the human *TLR4* gene, starting 4.3 kb upstream of the ATG start codon. SNPs were located at bp -4038, -3612, -3002, -2604, -2570, -2081, -2026 and -1607. B-C. Conservation of the human *TLR4* promoter compared to *Bos Taurus* and *Sus scrofa* (b) *Rattus norvegicus* and *Mus musculus* (c). Polymorphisms in the human consensus sequence were defined by comparison with the gene bank sequence (AF177765) and positions are indicated by ovals, numbered relative to the transcription start site in the human gene bank sequence. Filled ovals are located on conserved sequence blocks. D. Pair-wise linkage disequilibria (LD) were calculated, using VG2 software. LD was measured using r^2^ where the color scheme presents the degree of LD (range 0.0 through 1.0) where the maximum LD value (r^2^ = 1) corresponds to the upper boundary of the color spectrum. E. *In silico* predictions of transcription factor binding using TFSEARCH. Five of the eight SNPs were located in sequences with a high degree of homology to possible transcription factor binding motifs.

Figure 1B, C is based on BOV [31].

### Statistical analysis

Allele frequencies were calculated and tested for agreement with Hardy-Weinberg equilibrium using the |^2^-goodness-of-fit test with two degrees of freedom or Fisher's exact test. Case and control subjects were compared using the contingency |^2^ test for independence or Fisher's exact test depending on the numbers. Linkage disequilibria (LD) are displayed graphically using visual genotype (VG) (http://pga.gs.washington.edu/VG2.html). Haplotype frequencies were calculated using the Expectation-Maximation (EM) algorithm, implemented in PowerMarker. Expression levels were compared using GraphPad InStat for Windows (version 3.06; GraphPad Software). TFSEARCH was used for *in silico* predictions of transcription factor binding sites, with a default threshold score of 85.0 (http://www.cbrc.jp/research/db/TFSEARCH.html).

## Results

### I. *TLR4* promoter SNPs in the Swedish Population

#### Single nucleotide polymorphisms (SNPs) in the human *TLR4* promoter

We determined *TLR4* promoter and upstream region sequences by direct sequencing of amplified PCR products and identified eight sequence variants in the Swedish population, located at -4038, -3612, -3002 -2604, -2570, -2081, -2026 and -1607 bp (Figure 1A). Four sequence variants are novel (-4038, -3612, -3002, -2604), while four have been described (-2081 = rs10983755 and -1607 = rs10759932 in the NCBI SNP database and -1607, -2570 and -2026 in De Staercke *et al*. [32]). Six of the eight SNPs had minor allele frequencies >5% (Table S5), while SNPs -3002 and -2081 had minor allele frequency of 1% and 4%. Control markers for all genotypes were in Hardy-Weinberg equilibrium (χ^2^-test).

The overall conservation of *TLR4* upstream sequences was examined, by comparing the human reference sequence to known sequences from chimpanzee, cow, pig, mouse and rat. Conserved blocks showing at least 70% sequence identity to the human promoter were identified (Figures 1B,C and table S6). The chimpanzee promoter contained the eight sequence variants (Table S6) but the other species showed less homology, especially the mouse and rat (Figure 1B,C). Thus, even though *TLR4* is highly conserved from Drosophila to man, the region upstream of the proximal *TLR4* promoter has undergone substantial evolution in the mammalian lineage.

Pair-wise LDs analysis (Figure 1D) showed that only SNPs -4038 and -3612 were in complete LD (r^2^ = 1.00), indicating that they have not been separated by recombination or recurrent mutation. Furthermore, SNPs -2570 and -2026 were in strong LD (r^2^ = 0.92). However, most of the *TLR4* upstream promoter SNPs were not strongly linked indicating that this region may have undergone multiple recombination/mutation events and that gene conversion might have occurred during evolution.

*In silico* predictions of possible transcription factor binding site alterations were made, using TFSEARCH. Four of the eight SNPs were located in sequences with high homology to transcription factor binding motifs, while SNP -4038 and -2604 were suggested to form new binding sites (Figure 1E). SNP -4038 created and -2570-A/G potentially disrupted a binding site for CdxA, a homeodomain protein, and Tst-1, a POU (Pit-Oct-Unc) domain transcription factor which regulates early embryogenesis, neural development and late viral genes during glia-specific human papovavirus JC infection [33]. Variants -3612 and -3002 had no predicted effect on transcription factor binding sites, but SNP -2081 G/A disrupted a possible binding site for N-Myc, a basic MYC family helix-loop-helix transcription factor that regulates expression of many genes through Enhancer Box binding (E-boxes). SNP -2026 A/G potentially disrupted sites for Oct-1 and C/EBP, a CCAAT/enhancer protein. Oct-1, a POU domain transcription factor, regulates a variety of tissue-specific and general housekeeping genes by recruiting specialized transcriptional co-activators. -1607 T/C was predicted to abrogate binding of Nkx-2, a highly expressed homeobox transcription factor critical during early cardiac development [34]. According to TFSEARCH, conserved SNP -4038 and -2604 may form new transcription factor binding sites (Figure 1E). SNP -4038 created a potential CdxA site, while SNP -2604 formed a site for GATA-2, a transcription factor in hematopoietic cells that has been implicated as a negative regulator [35]. The *in silico* predictions thus suggest mechanisms by which the *TLR4* upstream region sequence variants could affect TLR4 protein expression.

#### *TLR4* promoter haplotype and genotype analysis

**Figure 2:**
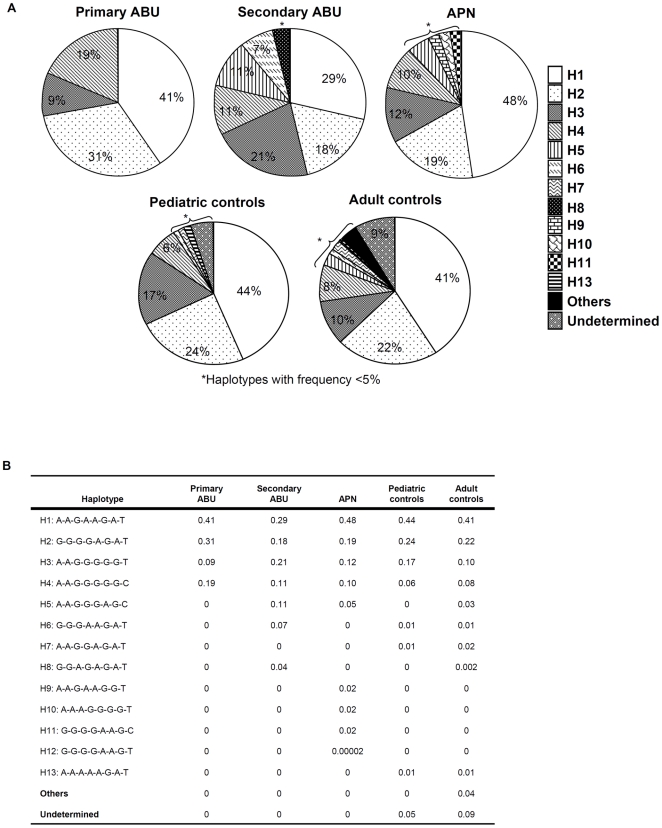
Haplotype analysis of *TLR4* promoter polymorphisms in pediatric UTI patients and controls. A. Circle diagram showing the haplotype frequency in the UTI groups and controls. The ABU groups differed significantly from the pediatric controls and between the ABU and the APN groups, but there was no difference between the two control groups or between the APN and controls. B. Haplotype frequency in patients and controls. H4 was more common in the primary and H5 in the secondary ABU patients. There was no difference haplotype frequency between APN and control groups.

We identified a total of 19 promoter haplotypes in the adult control population (Figure 2); H1-4 accounted for more than 80%, haplotypes 5 and 7 were found in 3% and 2%, respectively. The remaining haplotypes each occurred in less than 1% of the individuals.

**Figure 3:**
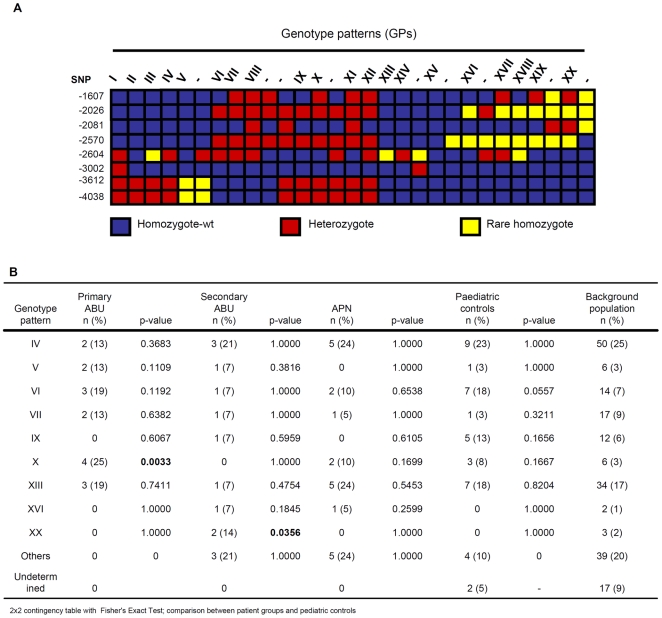
*TLR4* promoter genotypes. A. *TLR4* promoter GPs. Each column represents a genotype and each row a polymorphic site. Common allele homozygote (blue), heterozygote (red) and rare allele homozygote (yellow) are shown. Genotype patterns marked I-XX were found in ≥2 individuals, while GPs marked with – were found in one individual. B. GP frequencies in UTI prone patients and controls (Fisher's Exact Test). GPX was significantly more common in the primary ABU group and GPs V and VII were slightly more common in primary ABU, while GP XX was more common in secondary ABU. No difference in GP frequency was observed between the controls and the patients with APN but patients with APN group differed significantly from those with ABU.

Genotype patterns (GPs) were assigned by combining multiple SNPs in each individual (Figure 3A). Twenty combinations of multiple SNPs (GPs I-XX) were distinguished. GP IV was the most common (25%) followed by GP XIII (17%), GP VII (8.5%), GP VI (7%) and GP IX in 6% of the population (Figure 3B, table S7). The remaining GPs were present in less than 3% of the population, including GP II, the previously defined promoter sequence described in AF177765 (<1%).

### II. Disease associated TLR4 promoter SNPs in patients with UTI

Since TLR4 plays a vital role in UTI susceptibility, we examined *TLR4* promoter sequences in two groups of highly selected UTI prone patients with either a consistent pattern of asymptomatic carriage or APN, which is the most severe form of UTI. The patients were compared to pediatric and adult controls without UTI.

#### Study 1. *TLR4* promoter genotype variants in UTI-prone children

**Figure 4:**
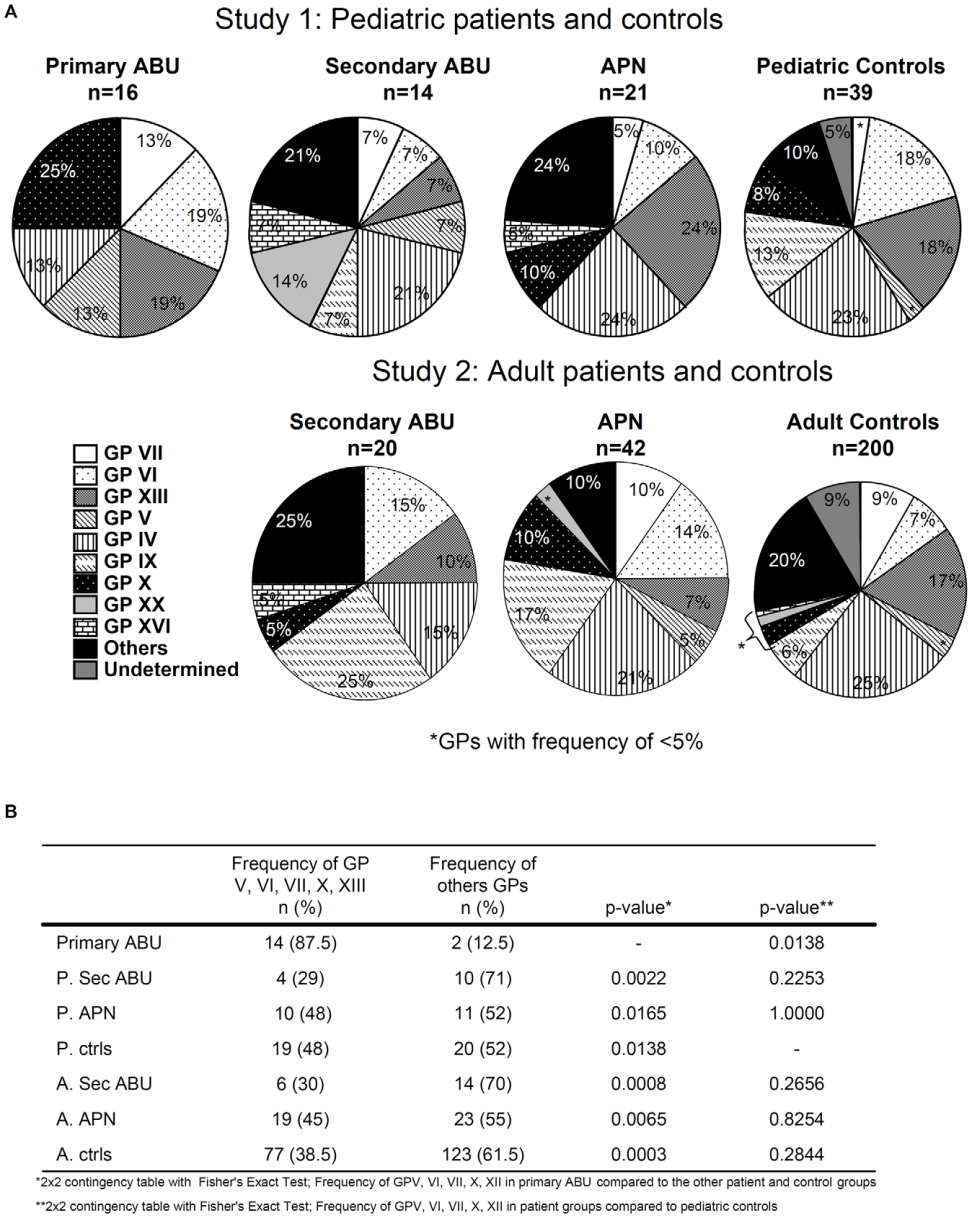
*TLR4* promoter genotype patterns (GPs) in UTI patient and control groups. A. GP distribution in pediatric (study 1) and adult (study 2) patients and controls. The UTI groups differed significantly compared to controls, but there was no difference between the pediatric and adult controls. B. The combined frequency of the most common GPs in primary ABU (GPs V, VI, VII, X, XIII) was compared to pediatric secondary ABU (p. sec ABU), pediatric APN (p. APN) and adult secondary ABU (a. sec ABU) or adult APN (a. APN)) groups.

This included children who either had a consistent pattern of asymptomatic carriage (primary ABU) or APN during at least 5 years of follow up. The *TLR4* promoter variability differed between asymptomatic carriers and controls or patients with severe disease. Only six of the eight detected SNPs were present (Table S4) in patients with primary ABU and this group had fewer haplotypes than the controls. Haplotype 4 was approximately 3x more common than the control groups (p = 0.0765; p = 0.0504), while the frequency of haplotypes 1, 2 and 3 did not differ significantly (Figure 2). The primary ABU patients had also a reduced number of GPs and the proportion of specific GPs differed compared to the background population (2×8 contingency table; p = 0.0007) (Figure 3, 4). GP X was significantly more common in the primary ABU group (Figure 2C, p = 0.0033), GPs V and VII were also more frequently found in the primary ABU group compared to the control populations (13% vs. 3% for both V and VII). GP IX, which accounted for 13% of pediatric controls, was absent in this group (Figure 3B, 4).

In contrast, the APN patient group carried the eight SNPs and did not differ in SNP frequency from pediatric controls, except for the heterozygote SNP -2081 (Table S4, p = 0.03). They carried four rare haplotypes (H9-H12) (p<0.05 and p = 0.0008 when compared to the control populations) but the frequency of GPs did not differ compared to controls (Figure 3B, 4). The patients with secondary ABU carried the eight SNPs; the rare -2570 homozygote, the rare homozygous at -1607 and SNP -2081 differed in frequency compared to pediatric controls (Table S4). This increase in rare homozygous variants was also visible in the haplotypes, which differed markedly between secondary ABU, pediatric and adult controls (Figure 2). H5 was more frequent in the secondary ABU group (11% vs. 0%; p = 0.0170). GP XX was exclusively found in secondary ABU (p = 0.0356) and GPs X and XIII were decreased. There was no significant difference in *TLR4* promoter haplotypes, genotype frequencies or GP distribution between adult and pediatric controls.

#### Study 2. *TLR4* promoter genotype variants in UTI-prone patients; 30 years follow up

To confirm the association between *TLR4* promoter genotype variants and UTI, we examined a second, highly UTI prone population. The patients had their first episode of febrile UTI in the 1970ies and were reinvestigated after about 30 years, including genetic analysis. The APN group was selected based on a consistent pattern of recurrent APN without ABU and carried the same eight *TLR4* promoter SNPs as APN patients in study 1. Their genotype frequency varied compared to controls at -2604 and -2570 (Table S4). Furthermore, GPIX was more common than in the control group (Table S8, p = 0.0113) and H3 was slightly more common (Figure S1). In the secondary ABU group no significant difference in genotype frequency was observed compared to the control group, apart from SNP-2570 which varied like the pediatric secondary ABU group. In addition, like in the APN group GPIX was found in higher frequency compared to the control population (p = 0.0113) (Figure 4, table S8). Furthermore, H3 was found in much higher frequency than in the adult controls (Figure S1, p<0.0001), similar trend was observed in the pediatric population (Figure S1).

The results propose a possible association between *TLR4* promoter sequence variants, promoter GPs and UTI severity. Furthermore, marked differences were observed between children with primary ABU and APN.

### III. TLR4 SNPs alter basic transcription efficiency and the response to infection

To examine whether the promoter sequence variants influence transcription efficiency, we constructed luciferase reporter plasmids carrying *TLR4* promoter sequences with single or multiple changes. The GPIV promoter (wildtype) was cloned and each of the eight SNPs was introduced by infusion cloning methodology. Human kidney cells were transfected either with the GPIV (wt) or each of the eight SNPs (single SNP constructs). Multiple SNPs representing the most common GPs were also introduced into the GPIV background (multiple SNP constructs) and transfected into human kidney cells. Promoter activity was determined as the luciferase activity relative to a renilla control plasmid.

**Figure 5:**
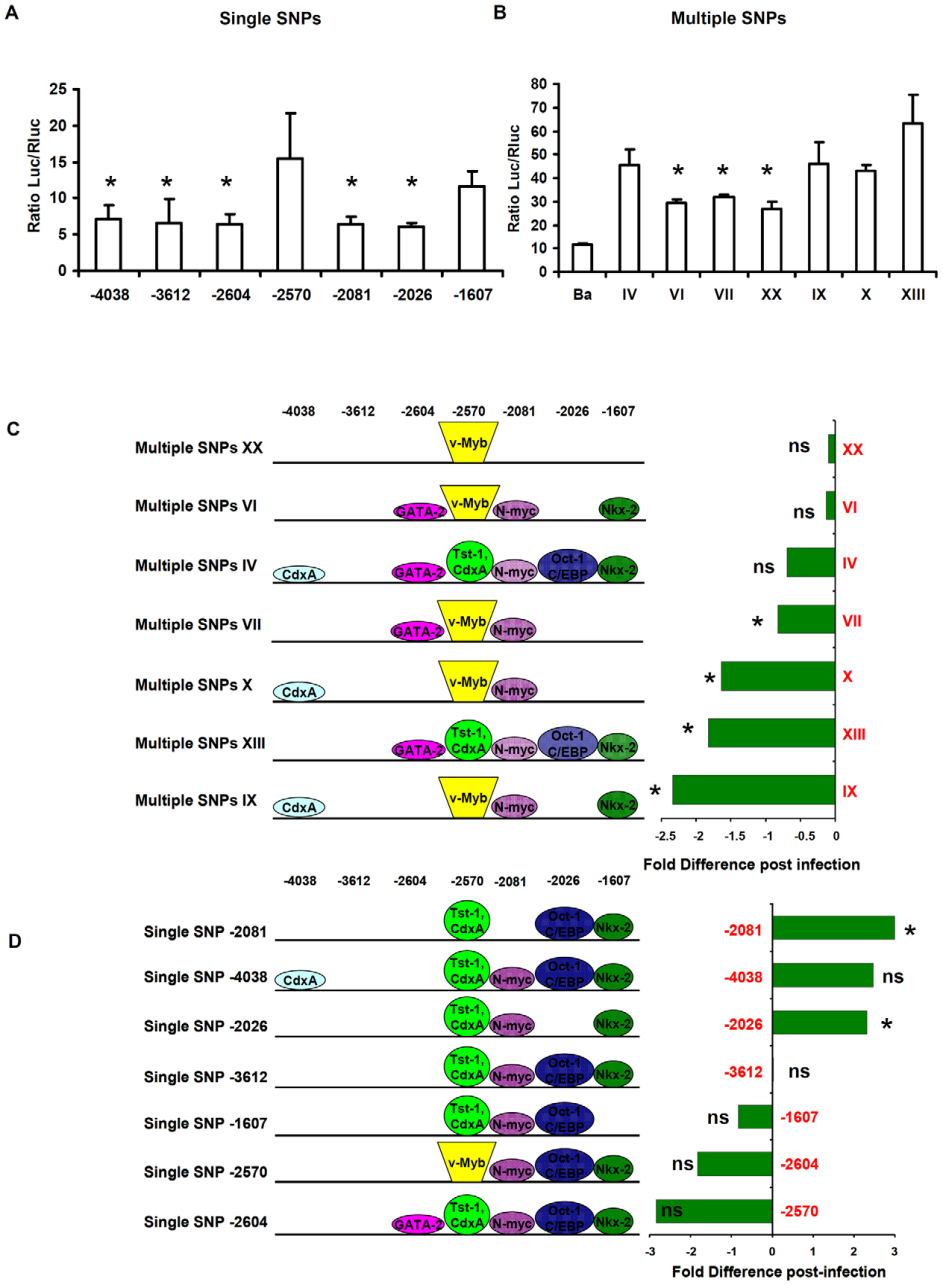
*TLR4* promoter GPs common in ABU patients reduce transcription efficiency. A-B. *TLR4* promoter activity determined in a luciferase reporter assay. Human renal carcinoma cells (A498) were transfected with plasmids carrying *TLR4* promoter sequences in frame of a luciferase reporter gene. The single SNP promoter constructs contained each of the detected SNPs. The multiple SNP constructs corresponding to GPs in the primary or secondary ABU groups. Luciferase levels were compared to GPIV, which was most common in the population and unrelated to UTI susceptibility. All transfected cells showed increased promoter activity compared to the background (Ba) control (means of three independent experiments, mean+/− SEMs). C-D. Change in promoter activity after infection with the uropathogenic *E. coli* strain CFT073 and schematic diagram showing possible transcription factor binding sites. The change in promoter activity after *E. coli* CFT073 infection is shown relative to the GPIV control. The histogram shows the fold difference in promoter activity post-infection for ABU-associated GPs and for the corresponding single SNPs. SNPs VII, X, XIII and IX significantly reduced promoter activity in response to infection. SNPs -2081 and -2026 significantly increased luciferase activity while -2604 and -2570 reduced the response to infection. (* = significant, ns = not significant, compared to uninfected cells carrying the same plasmids, Student T test).

The single and multiple SNP constructs were shown to modify luciferase expression levels. Five of the single SNP constructs differed significantly from the GPIV control (p<0.05), while two did not (-2570 and -1607) (Figure 5A). Primary ABU associated multiple SNP constructs VI, VII and XX showed significantly lower baseline TLR4 reporter expression levels, compared to IV (p<0.05, Figure 5B).

The change in promoter activity after infection with the virulent uro-pathogenic isolate *E. coli* CFT073 was examined by exposing transfected human kidney cells to *E. coli* CFT073 (4 h), and comparing luciferase levels before and after infection (Figure 5C and D). The single SNP constructs for -2081 and -2026 showed significantly higher luciferase reporter activity after stimulation with CFT073, but the others showed no significant change. For -2081 and -2026, TRANSFAC predicted loss of a potential transcription factor binding site; N-Myc for -2081 and Oct-1 and C/EBP for -2026. Multiple SNP constructs VII, IX, X and XIII significantly reduced reporter activity post infection. Interestingly, IX from the secondary ABU group showed the strongest decrease in luciferase response after infection.

*In silico* predicted changes in transcription factor binding resulting from the single or multiple SNPs are schematically represented in Figure 5C and D. The analysis identified complex relationships, potentially involving cross-modulation of repressive and enhancing SNPs. For example, repressive SNPs -2570 and -2604 which encode new transcription factor binding sites appear to negatively modulate each other as seen in GP VI and VII. SNP -4038, defined by acquisition of promoter activity, seems to modify the SNP -2604 repressor in GP IV but prevailed over by SNP -2570 in GPs X and IX. GP XX suggested that a consensus SNP -2081 sequence may act as a co-repressor for SNP -2604. However, further studies are needed to establish more precisely the nature of these complex relationships.

### IV. TLR4 promoter genotype and the innate immune response to human therapeutic urinary tract inoculation

**Figure 6:**
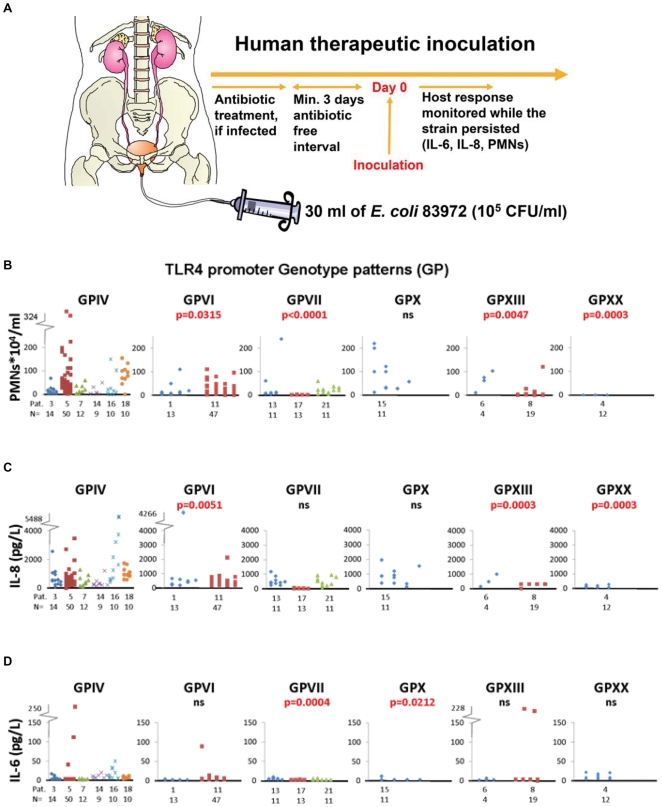
Primary ABU associated GPs lower innate immune response to therapeutic inoculation of UTI prone patients. (A) Patients (n = 15) were subjected to therapeutic intravesical inoculation with the prototype ABU strain *E. coli* 83972. The innate host response was quantified as the neutrophil numbers (B), IL-8 (C) and IL-6 (D) concentrations in urine at various times after inoculation. The response of patients with the ABU associated GPs (VI, VII, X, XIII) was compared to the most common and not UTI associated GPIV. Patients with the ABU associated GPs showed decreased responses compared to the GPIV controls. Pat.  =  Individual patient number. N = number of samples from each patient.

To examine if *TLR4* promoter GPs influence innate immune responses *in vivo*, in the human urinary tract, we determined the SNPs and GPs of patients who were subjected to therapeutic urinary tract inoculation with the ABU strain *E. coli* 83972 (Figure 6). The mucosal innate immune response to infection was quantified in urine as the IL-6 and IL-8 concentrations and as neutrophil (PMN) numbers. Responses were compared between patients with ABU associated promoter GPs and patients with GPIV; the common, not UTI associated control.

Patients with the primary ABU associated GPs (VII, X) showed lower innate immune response than the GPIV controls; patients with GPVII had lower neutrophil counts (p<0.0001) and IL-6 concentrations (p = 0.0004) and the patient with GPX had lower IL-6 levels (p = 0.0212). Furthermore, the patients carrying other GPs found in the primary ABU group (VI and XIII) had decreased IL-8 and PMN responses. GPVI and VII had low baseline *TLR4* promoter activities (Figure 4) and these GPs were strongly associated with asymptomatic status, relative to GPIV. Both GPs showed significant decrease in two of the three immune response parameters measured.

The results indicate that *TLR4* promoter variation may influence the mucosal response to *E. coli* infection.

## Discussion

The marked differences in susceptibility to common infections predict that there is substantial variation in the efficiency of the antimicrobial defense within the population. Yet, the mechanisms underlying exaggerated or attenuated host responses to many common infections remain unclear. As TLR4 is essential for innate immunity, understanding the genetic basis of varied TLR4 receptor expression and function is of great importance for the many biological endpoints that depend on TLR4 signaling. We describe a new concept for human TLR variation, based on *TLR4* promoter polymorphisms that influence expression dynamics *in vitro* and innate immune response dynamics in patients with ABU. The results suggest that reduced TLR4 expression attenuates the innate mucosal response, thus promoting an asymptomatic carrier state rather than severe disease.

We show that the *TLR4* promoter region is considerably more variable than previously known, in contrast to many other genes, where the overall variability is limited and closely positioned SNPs usually belong to the same or a small number of haplotypes. Interspecies comparisons of the *TLR4* promoter region confirmed this variability, as the sequences were poorly conserved except in the chimpanzee and a few blocks in human, cow and pig genomes. The few GPs in the primary ABU group might thus reflect selection for low responder variants, which protect from severe UTI, as shown in *Tlr4* ^-/-^ mutant mice, and possibly from other mucosal infections, where the host defense relies on TLR4 signaling.

The *TLR4* promoter is lacking TATA and CAT boxes and GC-rich motifs, which are typical for housekeeping gene promoters. Instead the *TLR4* promoter contains multiple binding sites for PU.1 and different *cis*-regulatory sequences may influence the transcription [36], [37]. Variations in expression levels have not been investigated, however. Previously, low TLR4 expression has mostly been attributed to tolerance and not to genetic variation affecting TLR4 expression. Michel *et al.* found no association between *TLR4* promoter polymorphisms and systemic inflammatory markers for LPS-induced systemic inflammation [38]. De Staercke *et al.* tried to link *TLR4* promoter polymorphisms to myocardial infarction risk but found no association [32]. This study is therefore the first to propose that genetic variation in the *TLR4* promoter influences TLR4 expression.

We also showed that single and multiple SNPs mostly suppressed TLR4 promoter activity *in vitro* and especially the response to *E. coli* infection. The mechanism of TLR4 activation by uropathogenic *E. coli* has been extensively studied in human cells and animal models [39]. Our early studies in C3H/HeJ mice showed that they developed ABU rather than APN, suggesting that a functional Tlr4 response is essential for inflammation and disease [19]. TLR4 was subsequently shown to control the response to infection of epithelial cells lining the urinary tract mucosa [21], [26]. While UPEC rely on many different mechanisms of host attack [40], TLR4 signalling is activated by P fimbrial binding to cell surface glycosphingolipid receptors [41] and ceramide acts as a signalling intermediate, activating TLR4 [42], [43]. In the human therapeutic inoculation model, P fimbriae were shown to promote the establishment of bacteriuria and mucosal inflammation [21], [26]. The observation that *TLR4* promoter sequence variation may influence the clinical presentation of UTI is consistent with these earlier studies.

For a long time, UTI susceptibility has mainly been discussed in terms of social and environmental factors or dysfunctional voiding [44], [45], [46], [47]. Now it is clear that genetic variation influences UTI susceptibility, determined by the severity of acute infection as well as the long-term effects on renal tissue integrity. Mice lacking the murine chemokine receptor 2 (m*Cxcr2* ^-/-^) develop APN with urosepsis and progressive renal scarring [15], [48]. The human correlate is reduced CXCR1 expression in APN prone patients, associated with heterozygous CXCR1 sequence variants [15], [16]. In contrast, there is no evidence that specific immune dysfunctions contribute to basic UTI susceptibility, in TCR mutant, RAG and SCID mice and there is no evidence of increased UTI susceptibility in patients with hypo-gammaglobulinemia or T cell deficiencies [49], [50]. Furthermore, Karoly *et al.* showed that patients with UTI had higher prevalence of *TLR4* SNP Asp299Gly than controls (p = 0,041). The SNP tended to occur more frequently in patients with recurrent UTI without VUR than in patients with vesicoureteral abnormalities (p = 0,067) [51]. Hawn *et al.* suggested that Asp299Gly was associated with protection from recurrent UTI, but not pyelonephritis. Furthermore, they showed that a *TLR*5_(C1174T) polymorphism was associated with an increased risk of recurrent UTI but not pyelonephritis, while a polymorphism in *TLR*1_(G1805T) was associated with protection from pyelonephritis [52]. Furthermore, a recent study by the same group reported that a *TLR*2_(G2258A) polymorphism, which has been associated with decreased lipopeptide-induced signaling, was associated with increased ABU risk [53]. This current study adds *TLR4* promoter sequence variation to the list of human genetic variants influencing UTI susceptibility, further emphasizing the importance of innate immune regulation in UTI.

The present study also proposes that distinct genetic traits might distinguish asymptomatic carriers from patients with symptomatic UTI. While we are aware that genetic association analyses usually are based on thousands of samples, a different approach was taken. We used long-term follow up combined with selection to define highly disease prone individuals. The two unique long-term follow-up protocols both started in childhood, when UTI susceptibility is diagnosed and lasted until it was possible to distinguish patients with a clear pattern of APN from those who developed ABU. This approach allowed us to observe significant differences between relatively small groups of patients with different forms of UTI, indicating that the patient material is informative. This level of clinical definition is quite demanding, but in suitable translational environments where candidate genes are identified, this approach might prove useful to assess if such genes may contribute to disease susceptibility.

The challenges for the innate immune system at mucosal surfaces differ from those at systemic sites after bacterial invasion. While pathogens are recognized and often defeated by the antibacterial defense, asymptomatic carriage is the most common outcome of host-parasite interaction, creating the type of symbiosis seen with the commensal microflora. Asymptomatic *E. coli* carriage is very common, occurring in 1% of girls, >2% of pregnant women and >10% of elderly individuals [54], [55], [56]. ABU has been shown to protect against infection with more virulent bacteria [57], explaining why most clinical centers leave ABU untreated. These observations have given rise to the use of human therapeutic inoculation [26], which has been found safe and efficient in open clinical studies and more recently in a placebo-controlled study (Sundén *et al*., In press in Journal of Urology). It was therefore possible for us to examine if the innate immune response in the urinary tract of several patients receiving *E. coli* 83972 may reflect their *TLR4* promoter genotypes. The results supported this hypothesis, thus providing unique human data suggesting that *TLR4* promoter variation may be a significant human innate immune variable.

## Supporting Information

Figure S1 Haplotype analysis of TLR4 promoter polymorphisms in adult UTI patients and controls A. Difference in haplotype distribution between adult UTI prone patients and controls. B. Frequency of each haplotype in patient and controls groups.
(2.60 MB TIF)

Table S1 Clinical characteristics of patients and controls included in this study.
(0.05 MB DOC)

Table S2 Primers used for amplification and sequencing of the TLR4 promoter and for pyrosequencing of TLR4 promoter SNPs.
(0.07 MB DOC)

Table S3 Primers used for construction of the different TLR4 promoter vectors used in transient transfections and dual luciferase reporter system assay.
(0.06 MB DOC)

Table S4 Genotype frequencies of TLR4 promoter SNPs.
(0.10 MB DOC)

Table S5 Allele frequencies of TLR4 promoter SNPs.
(0.08 MB DOC)

Table S6 Species specific sequence variation in the TLR4 promoter.
(0.07 MB DOC)

Table S7 Effects of TLR4 promoter SNPs on transcription factor binding sites in each GP as predicted by TFSEARCH.
(0.08 MB DOC)

Table S8 Genotype Pattern (GP) frequency in adult UTI prone patients and UTI free controls.
(0.06 MB DOC)
