# Supplementary figures and images for: Toll-Like Receptor 4 Promoter Polymorphisms: Common TLR4 Variants May Protect against Severe Urinary Tract Infection
Source: PLoS One. 2010 May 20;5(5):e10734. doi: 10.1371/journal.pone.0010734 (PMC2873976; doi:10.1371/journal.pone.0010734)

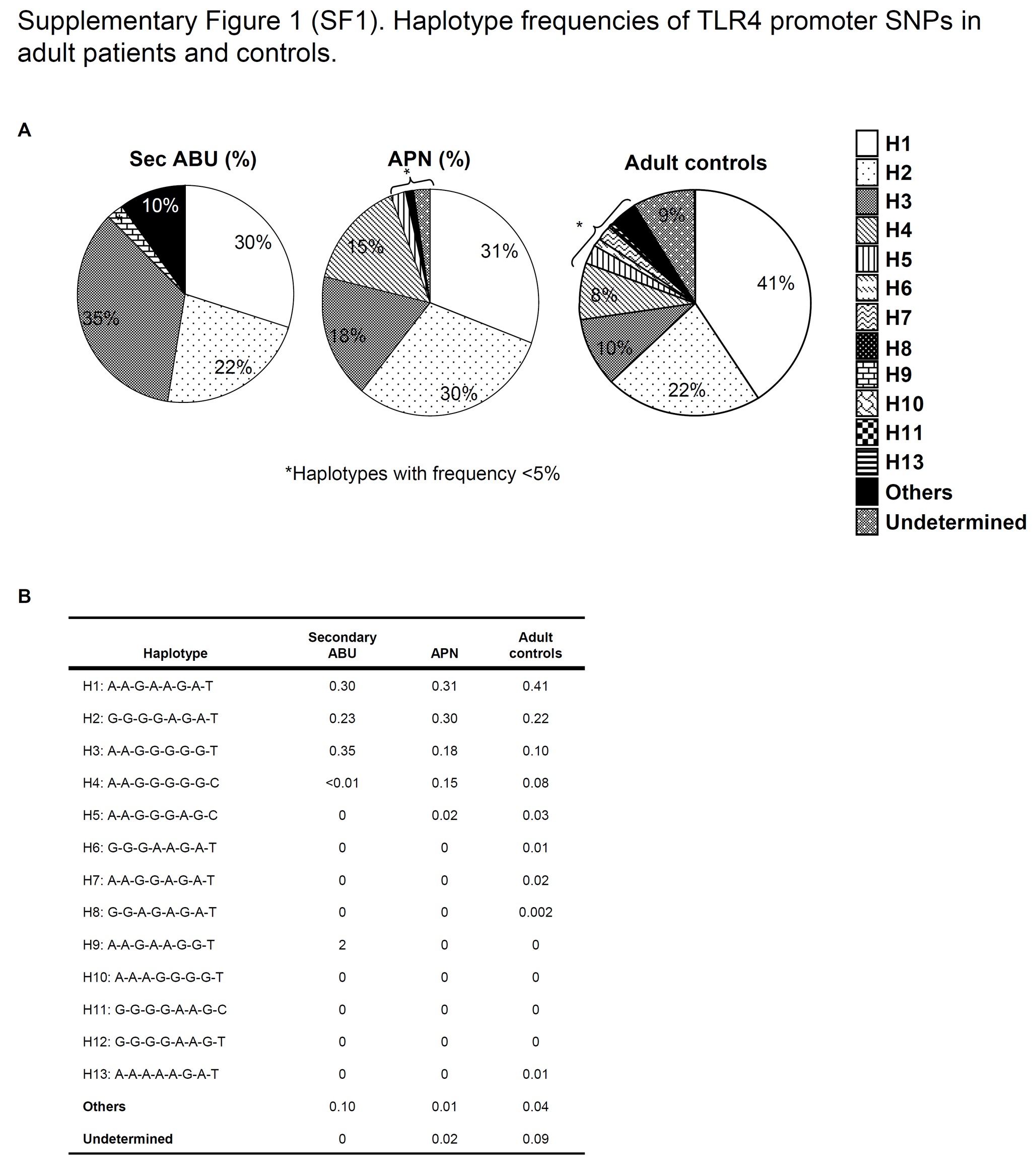

Supplement: Figure S1 — Haplotype analysis of TLR4 promoter polymorphisms in adult UTI patients and controls A. Difference in haplotype distribution between adult UTI prone patients and controls. B. Frequency of each haplotype in patient and controls groups. (2.60 MB TIF) [file pone.0010734.s001.tif]
