# Supplementary material for: Toll-Like Receptor 4 Promoter Polymorphisms: Common TLR4 Variants May Protect against Severe Urinary Tract Infection
Source: PLoS One. 2010 May 20;5(5):e10734. doi: 10.1371/journal.pone.0010734 (PMC2873976; doi:10.1371/journal.pone.0010734)
Supplement: Table S1 — Clinical characteristics of patients and controls included in this study. (0.05 MB DOC) [file pone.0010734.s002.doc]

**Table S1.** Clinical characteristics of patients and controls included in this study

| **Patient/control group** | **Total number of patients** | **Sex F/M** | **Median age years**  **(range)** | **Clinical characteristics** | **Comment** |
| --- | --- | --- | --- | --- | --- |
| **Study 1 (N=90): pediatric patients and controls** | | | | | |
| Primary ABU^a^ | 16 | 9/7 | 5 (<1-18) | These patients did not have symptomatic UTI during a follow-up period of 6 years (2001-2007) | A diagnosis of ABU was based on ≥ 3 consecutive urine cultures yielding the same bacterial strain (>10^5^ CFU/mL urine) in a child with no symptoms of UTI and no elevation of CRP |
| Secondary ABU^a^ | 14 | 10/4 | 7 (1-13) | These patients had one or more episodes of symptomatic UTI before or after developing ABU during a follow-up period of 6 years. |  |
| Acute pyelonephritis^a^ | 21 | 18/3 | 8.5 (1-13) | These patients did not develop ABU at any time during a follow-up period of 6 years. | The diagnosis was based on a febrile infection (≥38.5°C), significant bacteriuria, elevated C-reactive protein >20 mg/l and lack of symptoms of other infections**.** |
| **Study 2 (N=262): Adults with history of symptomatic UTI during childhood and adult controls** | | | | | |
| Acute pyelonephritis^b^ | 42 | 42/0 | 4 (<1-15)^c^ | These patients had febrile UTI in the 1970s, and were reinvestigated with a median of 30 years after the initial UTI episode.  The patients in the APN group had a debut APN diagnosis, first recidive APN infection or multiple APN episodes during the 30 year period. | Patients were reinvestigated to evaluate genetic associations between UTI morbidity and long-term effects of these infections on health and kidney function. |
| Secondary ABU^b^ | 20 | 20/0 | 8 (<1-14)^c^ | These patients had febrile UTI in the 1970s, and were reinvestigated with a median of 30 years after the initial UTI episode.  These patients had one or more episodes of symptomatic UTI before or after developing ABU. | Patients were reinvestigated to evaluate genetic associations between UTI morbidity and long-term effects of these infections on health and kidney function. |
| Pediatric controls^a^ | 39 | 10/29 | 6 (<1-18) | No symptoms from the urinary tract. Negative urine cultures at the time of sampling. No history of UTI. | Enrolled in the pediatric outpatient clinic or admitted for elective surgery for diagnoses unrelated to infection. |
| Blood donors^a^ | 200 | Unknown | Adults | Healthy at sampling |  |
| **Therapeutic inoculation (N=15)** | | | | | |
| Therapeutic inoculation^a^ | 15 | 7/8 | Adults | Neurogenic bladder disorders and a history of recurrent lower UTI | Patients participated in a placebo controlled study demonstrating therapeutic efficacy of the human colonization protocol. |

a, treated or sampled at Lund University Hospital

b, treated at the Queen Silvia Children’s Hospital in Gothenburg

c, debut age of symptomatic UTI
