# Supplementary material for: Toll-Like Receptor 4 Promoter Polymorphisms: Common TLR4 Variants May Protect against Severe Urinary Tract Infection
Source: PLoS One. 2010 May 20;5(5):e10734. doi: 10.1371/journal.pone.0010734 (PMC2873976; doi:10.1371/journal.pone.0010734)
Supplement: Table S2 — Primers used for amplification and sequencing of the TLR4 promoter and for pyrosequencing of TLR4 promoter SNPs. (0.07 MB DOC) [file pone.0010734.s003.doc]

**Table S2.** Primers used for amplification and sequencing of the *TLR4* promoter and for pyrosequencing of *TLR4* promoter SNPs.

A. Amplifying PCR primers

| **Name** | **Forward (5’-3’)** | **Reverse (5’-3’)** |
| --- | --- | --- |
| **TLR4-1A** | TGCAGTAAACTTGGAGGCTG | GACCCCATTACTGGGACACA |
| **TLR4-1Ab** | CCTACTTTCTTCACATTCTGC | GAGAGGAAACCGCTTGATAG |
| **TLR4-2A** | TAGGAAAAATTTCCAGGAAGA | GAGACTGCACTTACCAGGGCTA |
| **TLR42Ab** | GCTTACAGTTTCTAATTTGTATTTG | GAGGTTATTCAAACTAGCCAGTC |
| **TLR4-3A** | GCAGAAAAGGCAAGGCAAGGC | CTTTAAAACGAAGATAATCGC |
| **TLR4-3Ab** | CAACAGATCAGAAGATGATGC | GCAGTGCCTGACACAGAGTAGG |
| **TLR4-4A** | CCAGTTCATTTAATCCCAATA | CGTACAAGAAGATTGGGAAAAG |
| **TLR4-5A** | GAATAGTGGTGATGATTGTAC | GGCAGACATCATCCTGGCATC |
| **TLR4-6A** | GTATGTGAGTTTCTTCACAAG | GAAAGTTCAGAGGAGCTGACTC |

B. Nested PCR primers

| **Name** | **Forward (5’-3’)** | **Reverse (5’-3’)** |
| --- | --- | --- |
| **TLR4-1N** | ATGTTGAATATGAAAGTATAATG | GAATAGAGTAATACAAGTGTGT |
| **TLR4-1Nb** | GGAGGCTGCATGTTGAATATG | GTCCTTTCAACTATGTTTTCG |
| **TLR4-2N** | AACACATGTATTAACATCTCCG | GATTTGCTCTGGAGCATGCCTT |
| **TLR42Nb** | GGTCTGCCTTCTGGAAGAGCAGC | GCTTACCCTGCCTTGCCTTGCC |
| **TLR4-3N** | TAAGCAGGGATAGGACTGGC | GAATTAAGCTAACTTACGTA |
| **TLR4-3Nb** | GCCACATTGGTAGCACCAGAG | GTTAATGGTGTTTCACTTTGAC |
| **TLR4-4N** | GATACATATTATCATCTTCATTC | GGAATATACTGAAGTTCAGATC |
| **TLR4-5N** | TATGAACATAATTAATGCCAC | CTCACTGCTTCTGTGAGCAGCA |
| **TLR4-6N** | CCTACATATCGAAGTCCTAAC | CCACATACCTCCACGCAGGGC |

C. Amplifying primers and sequencing primers for pyrosequencing

| **SNP** | **Forward (5’-3’)** | **Reverse (5’-3’)** | **Sequencing (5’-3’)** |
| --- | --- | --- | --- |
| **-4038** | CCTGCCAAATAAAAGCAAACAC | CAGTCAATGGAGGAATCCCATAT-BIO | TTCTAGGTCCCTGGC |
| **-3612** | TTTGTATTTGACACATGGTCTGC | TGGGAAATGAATGGGATTAACAC-BIO | CATTACTATTGAACATATCC |
| **-3002** | BIO-AATCAATTGGAAGAGCTGGTACA | CAGTCGCCATTTCTACTACCATTA | CATTACTATTGAACATATCC |
| **-2604** | BIO-TCAGTGGGCTCTGGGGTAG | CAGCCCTAATCATCACAGGTC | CATCACAGGTCCAGGTA |
| **-2570** | BIO-TGGTACCTGGACCTGTGATGAT | CCTCCTCTACCTGGCTTTTACA | GCTTTTACACCCAAGTAGAC |
| **-2081** | TACAAGAGTTTGTGCCCAGTCCA | GCAAGTGCAATGTAAGTTTCTGTT-BIO | CCCTCACAGCTTGGTT |
| **-2026** | TTGGAAGTGCTTGGAGGATATTA | TTAGGACAGTGTCTGGAAAGTAGC-BIO | AGAACTATCTAGGACTTAGC |
| **-1607** | BIO-AAATGCAAGCTTCTGCTATGATTA | TCAGAAGTGAGATTGCTGGATCAT | TTTCACATCTTCACCAAC |
