# Supplementary material for: Toll-Like Receptor 4 Promoter Polymorphisms: Common TLR4 Variants May Protect against Severe Urinary Tract Infection
Source: PLoS One. 2010 May 20;5(5):e10734. doi: 10.1371/journal.pone.0010734 (PMC2873976; doi:10.1371/journal.pone.0010734)
Supplement: Table S3 — Primers used for construction of the different TLR4 promoter vectors used in transient transfections and dual luciferase reporter system assay. (0.06 MB DOC) [file pone.0010734.s004.doc]

**Table S3**. Primers used for construction of the different *TLR4* promoter vectors used in transient transfections and dual luciferase reporter system assay

A. Primers used for adding SNPs

| **SNP** | **Primer sequence** |
| --- | --- |
| **SNP -4038:** G to A | CAC TTC TAG GTC CCT G GC **A**AA TAT GGG ATT CCT CCA TTG ACT G |
| **SNP -3612:** G to A | GGT CTG CCT TCT GGA AGA GCA **A**CA TAG AAC CTA GAT GTC |
| **SNP -2604:** G to A | TCT CTA GTT GTC TG**A** TAC CTG GAC CTG TG |
| **SNP -2570:** A to G | TAG GGC TGA ATA AC**G** GTG TCT ACT TGG GT |
| **SNP -2081:** G to A | CAG CTT GGT TTT TGA CAC **A**TT GGA TTG GAA GTG CTT GG |
| **SNP -2026:** A to G | CTA GGA CTT AGC AT**G** CAT AAT ATT CCT GT |
| **SNP -1607:** T to C | AAA GTA ATA ATA AG**C** GTT GGT GAA GAT GT |

B. Primers for adding SNPs

| **SNP** | **Primer sequence** |
| --- | --- |
| - **4038** **original G** | GCA AA C ACT TCT AGG TCC CTG GC**G** AAT ATG GGA TTC CTC CAT TGA C |
| - **3612** **original G** | GGT CTG CCT TCT GGA AGA GCA **G**CA TAG AAC CTA GAT GTC |
| - **2604** **original** **G** | GGT CTG TCT CTA GTT GTC TG**G** TAC CTG GAC CTG TGA TG |

C. Sequencing primers for verification of constructs

| **Name** | **Primer sequence** |
| --- | --- |
| **F1A** | TGC AGT AAA CTT GGA GGC TG |
| **R1A** | GAC CCC ATT ACT GGG ACA CA |
| **R3A** | CTT TAA AAC GAA GAT AAT CGC |
| **F4A** | CCA GTT CAT TTA ATC CCA ATA |

D. Cloning scheme for the different multiple constructs

| **Construct** | **Template** | **Primers used to create SNPs** | **Primers used for removing SNPs** |
| --- | --- | --- | --- |
| VI | Blanc | -2604 G to A  -2570 A to G  -2026 A to G |  |
| VII | VI | -1607 T to C |  |
| IX | VI | -3612 G to A  -4038 G to A | -2604 A to G |
| X | IX | -1607 T to C |  |
| XIII | Blanc | -2604 G to A |  |
| XX | VI | -2081 G to A  -1607 T to C | -2604 A to G |

* Blanc: Template where all the SNPs had been removed from the initial TLR4 pGL 3 clone IV
