# Supplementary material for: Toll-Like Receptor 4 Promoter Polymorphisms: Common TLR4 Variants May Protect against Severe Urinary Tract Infection
Source: PLoS One. 2010 May 20;5(5):e10734. doi: 10.1371/journal.pone.0010734 (PMC2873976; doi:10.1371/journal.pone.0010734)
Supplement: Table S4 — Genotype frequencies of TLR4 promoter SNPs. (0.10 MB DOC) [file pone.0010734.s005.doc]

**Table S4**. Genotype frequencies of *TLR4* promoter SNPs

|  |  |  | **Paediatric patients and controls** | | | **Adult patients and controls** | | |
| --- | --- | --- | --- | --- | --- | --- | --- | --- |
| **SNP** | **SNP** | **Primary ABU** | **Secondary ABU** | **APN** | **Controls** | **Secondary ABU** | **APN** | **Controls** |
| **-4038** | A/A | 8 (50) | 7 (50) | 12 (57.1) | 19 (48.7) | 8 (40.0) | 18 (42.9) | 107 (53.8) |
|  | G/A | 6 (37.5) | 6 (42.9) | 9 (42.9) | 18 (46.2) | 12 (60.0) | 22 (52.4) | 83 (41.7) |
|  | G/G | 2 (12.5) | 1 (7.1) |  | 2 (5.1) | - | 2 (4.8) | 9 (4.5) |
|  | P-value | 0.5927 | 0.9503 | 0.5227 | 0.8458 | 0.2274 | 0.4259 | - |
| **-3612** | A/A | 8 (50) | 7 (50) | 12 (57.1) | 19 (48.7) | 8 (40.0) | 18 (43.9) | 107 (54.3) |
|  | G/A | 6 (37.5) | 6 (42.9) | 9 (42.9) | 18 (46.2) | 12 (60.0) | 21 (51.2) | 82 (41.6) |
|  | G/G | 2 (12.5) | 1 (7.1) |  | 2 (5.1) | - | 2 (4.9) | 8 (4.1) |
|  | P-value | 0.5927 | 0.9503 | 0.5227 | 0.8041 | 0.2311 | 0.4780 |  |
| **-3002** | G/G | 16 (100) | 13 (92.9) | 20 (95.2) | 38 (97.4) | 19 (95.0) | 41 (97.6) | 197 (98.5) |
|  | G/A | - | 1 (7.1) | 1 (4.8) | 1 (2.6) | 1 (5.0) | 1 (2.4) | 3 (1.5) |
|  | A/A | - | - | - |  | - | - |  |
|  | P-value | 1.0000 | 0.4623 | 1.0000 | 0.5120 | 0.3189 | 0.5359 |  |
| **-2604** | G/G | 6 (37.5) | 7 (50) | 6 (28.8) | 11 (28.2) | 8 (40.0) | 19 (45.2) | 49 (25.0) |
|  | G/A | 7 (43.7) | 6 (42.9) | 9 (42.9) | 19 (48.7) | 10 (50.0) | 20 (47.6) | 103 (52.6) |
|  | A/A | 3 (18.8) | 1 (7.1) | 6 (28.8) | 9 (23.1) | 2 (10.0) | 3 (7.1) | 44 (22.4) |
|  | P-value | 0.7891 | 0.2343 | 0.8755 | 0.8920 | 0.2357 | 0.0099 |  |
| **-2570** | A/A | 7 (43.7) | 6 (42.9) | 11 (52.4) | 21 (53.8) | 5 (25.0) | 15 (35.7) | 108 (56.3) |
|  | A/G | 9 (56.3) | 4 (28.6) | 8 (38.1) | 17 (43.6) | 13 (65.0) | 23 (54.8) | 64 (33.3) |
|  | G/G | - | 4 (28.6) | 2 (9.5) | 1 (2.6) | 2 (10.0) | 4 (9.5) | 20 (10.4) |
|  | P-value | 0.5619 | 0.0164 | 0.4900 | 0.2014 | 0.0156 | 0.0295 |  |
| **-2081** | G/G | 16 (100) | 11 (78.6) | 18 (85.7) | 39 (100) | 20 (100.0) | 39 (92.9) | 183 (92.4) |
|  | G/A | - | 4 (21.4) | 3 (14.3) |  | - | 3 (7.1) | 14 (7.1) |
|  | A/A | - | - | - |  | - | - | 1 (0.5) |
|  | P-value | - | 0.0043 | 0.0389 | 0.1406 | 0.3712 | 1.0000 |  |
| **-2026** | A/A | 7 (43.7) | 6 (42.9) | 9 (42.9) | 20 (51.3) | 7 (35.0) | 15 (35.7) | 108 (54.8) |
|  | A/G | 9 (56.3) | 4 (28.6) | 9 (42.9) | 16 (41.0) | 11 (55.0) | 23 (54.8) | 71 (36.0) |
|  | G/G | - | 4 (28.6) | 3 (14.3) | 1 (2.6) | 2 (10.0) | 4 (9.5) | 18 (9.1) |
|  | P-value | 0.5587 | 0.0209 | 0.2296 | 0.3631 | 0.2135 | 0.0625 |  |
| **-1607** | T/T | 10 (62.5) | 9 (64.3) | 15 (71.4) | 34 (87.2) | 17 (85.0) | 26 (65.0) | 149 (75.3) |
|  | C/T | 6 (37.5) | 4 (28.6) | 5 (23.8) | 5 (12.8) | 3 (15.0) | 12 (30.0) | 47 (23.7) |
|  | C/C | - | 1 (7.1) | 1 (4.8) |  | - | 2 (5.0) | 2 (1.0) |
|  | P-value | 0.0615 | 0.0839 | 0.1958 | 0.2511 | 0.5965 | 0.1253 |  |
