# Supplementary material for: Toll-Like Receptor 4 Promoter Polymorphisms: Common TLR4 Variants May Protect against Severe Urinary Tract Infection
Source: PLoS One. 2010 May 20;5(5):e10734. doi: 10.1371/journal.pone.0010734 (PMC2873976; doi:10.1371/journal.pone.0010734)
Supplement: Table S5 — Allele frequencies of TLR4 promoter SNPs. (0.08 MB DOC) [file pone.0010734.s006.doc]

**Table S5**. Allele frequencies of *TLR4* promoter SNPs

| **Patients and controls** |  | **-4038 (GG wt)** | **-3612 (GG wt)** | **-3002 (GG wt)** | **-2604 (GG wt)** | **-2570 (AA wt)** | **-2081 (GG wt)** | **-2026 (AA wt)** | **-1607 (TT wt)** |
| --- | --- | --- | --- | --- | --- | --- | --- | --- | --- |
| **Primary ABU** | p | 0.69 | 0.69 | 1.00 | 0.59 | 0.72 | 1.00 | 0.72 | 0.81 |
|  | q | 0.31 | 0.31 |  | 0.41 | 0.28 |  | 0.28 | 0.19 |
|  | p-value | 0.6418 | 0.6473 | 1.0000 | 0.5355 | 0.8099 | - | 0.8038 | 0.0800 |
| **Secondary ABU** | p | 0.71 | 0.71 | 0.96 | 0.64 | 0.57 | 0.89 | 0.57 | 0.79 |
|  | q | 0.29 | 0.29 | 0.04 | 0.36 | 0.43 | 0.11 | 0.43 | 0.21 |
|  | p-value | 1.0000 | 1.0000 | 0.4604 | 0.1183 | 0.0896 | 0.0170 | 0.2029 | 0.0648 |
| **APN** | p | 0.79 | 0.79 | 0.98 | 0.50 | 0.71 | 0.93 | 0.67 | 0.83 |
|  | q | 0.21 | 0.21 | 0.02 | 0.50 | 0.29 | 0.07 | 0.33 | 0.17 |
|  | p-value | 0.5141 | 0.5141 | 1.0000 | 0.8492 | 0.6647 | 0.0409 | 0.1871 | 0.1122 |
| **Paediatric controls** | p | 0.72 | 0.72 | 0.99 | 0.53 | 0.76 | 1.00 | 0.72 | 0.94 |
|  | q | 0.28 | 0.28 | 0.01 | 0.47 | 0.24 |  | 0.23 | 0.06 |
|  | p-value | - | - | - | - | - | - | - | - |
| APN | p | 0.69 | 0.70 | 0.99 | 0.69 | 0.63 | 0.96 | 0.63 | 0.80 |
|  | q | 0.31 | 0.30 | 0.01 | 0.31 | 0.37 | 0.04 | 0.37 | 0.20 |
|  | p-value | 0.3398 | 0.3316 | 0.5347 | 0.0036 | 0.0838 | 1.0000 | 0.0847 | 0.1118 |
| Sec. ABU | p | 0.7 | 0.7 | 0.975 | 0.65 | 0.575 | 1 | 0.625 | 0.925 |
|  | q | 0.3 | 0.3 | 0.025 | 0.35 | 0.425 | 0 | 0.375 | 0.075 |
|  | p-value | 0.5699 | 0.4523 | 0.3179 | 0.1339 | 0.0446 | 0.3818 | 0.1958 | 0.4520 |
| Adult CTRL | p | 0.74 | 0.75 | 0.99 | 0.51 | 0.73 | 0.96 | 0.73 | 0.87 |
|  | q | 0.26 | 0.25 | 0.01 | 0.49 | 0.27 | 0.04 | 0.27 | 0.13 |
|  | p-value | 0.6723 | 0.6697 | 0.5108 | 0.9014 | 0.6752 | 0.0867 | 0.7627 | 0.1730 |

Fisher’s Exact Test. 2x2 comparison between patient groups and paediatric controls
