# Supplementary material for: Toll-Like Receptor 4 Promoter Polymorphisms: Common TLR4 Variants May Protect against Severe Urinary Tract Infection
Source: PLoS One. 2010 May 20;5(5):e10734. doi: 10.1371/journal.pone.0010734 (PMC2873976; doi:10.1371/journal.pone.0010734)
Supplement: Table S6 — Species specific sequence variation in the TLR4 promoter. (0.07 MB DOC) [file pone.0010734.s007.doc]

**Table S6.** Species specific sequence variation in the *TLR4* promoter

|  | **Conserved sequences** |
| --- | --- |
| Cons | GAATATG |
| Variant -4038 | AAATATG |
| Chimpanzee | AAATATG (-1064) |
| Rat | AAATATG (-2936) |
| Mouse | AAATATG (-4252; 4093; -1460; -461) |
| Cons | AGAGCAGCATAGAA |
| Variant -3612 | AGAGCAACATAGAA |
| Chimpanzee | AGAGCAACATAGAA (-3616) |
| Cons | TAGGCCGTGGGATA |
| Variant -3002 | TAGGCCATGGGATA |
| Chimpanzee | TAGACCGTGGGATA (-3016) |
| Rat | ATGGGATA (-3597) |
| Mouse | GTGGGATA (-1954) |
| Cons | TCTGGTA |
| Variant -2604 | TCTGATA |
| Chimpanzee | TCTGGTA (-2614) |
| Cow | TCTGGTA (-3725) |
| Rat | TCTGATA (-2254) (-3062) |
| Cons | GAATAACAGTGTCTAC |
| Variant -2570 | GAATAACGGTGTCTAC |
| Chimpanzee | GAATAATGGTGTCTAC (-2586) |
| Cons | GACACG |
| Variant -2081 | GACACA |
| Chimpanzee | GACACG (-2093) |
| Cow | GACACG (-1416;-266) |
| Mouse | GACACG (-543) |
| Cons | CTTAGCATACA |
| Variant -2026 | CTTAGCATGCA |
| Chimpanzee | CTTAGCACGCA (-2044) |
| Cons | ATAAGTG |
| Variant -1607 | ATAAGCG |
| Chimpanzee | ATAAGTG (-1619) |
| Rat | ATAAGTG (-1591) |
