# Supplementary material for: Toll-Like Receptor 4 Promoter Polymorphisms: Common TLR4 Variants May Protect against Severe Urinary Tract Infection
Source: PLoS One. 2010 May 20;5(5):e10734. doi: 10.1371/journal.pone.0010734 (PMC2873976; doi:10.1371/journal.pone.0010734)
Supplement: Table S7 — Effects of TLR4 promoter SNPs on transcription factor binding sites in each GP as predicted by TFSEARCH. (0.08 MB DOC) [file pone.0010734.s008.doc]

**Table S7.** Effects of *TLR4* promoter SNPs on transcription factor binding sites in each GP as predicted by TFSEARCH

|  |  | **Predicted transcription factor binding sites present in each genotype pattern** | | | | | |
| --- | --- | --- | --- | --- | --- | --- | --- |
|  |  | **SNP** | | | | | |
| **Genotype** | **Population frequency (%)** | **-4038** | **-2604** | **-2570** | **-2081** | **-2026** | **-1607** |
| **I** | 1 | CdxA | GATA-2 | Tst-1, CdxA | N-Myc | Oct-1, C/EBP | Nkx-2 |
| **II** | 1 | CdxA | - | Tst-1, CdxA | N-Myc | Oct-1, C/EBP | Nkx-2 |
| **III** | 2 | CdxA | GATA-2 | Tst-1, CdxA | N-Myc | Oct-1, C/EBP | Nkx-2 |
| **IV** | 25 | CdxA | GATA-2 | Tst-1, CdxA | N-Myc | Oct-1, C/EBP | Nkx-2 |
| **V** | 3 | CdxA | - | Tst-1, CdxA | N-Myc | Oct-1, C/EBP | Nkx-2 |
| **VI** | 57 | - | GATA-2 | v-Myb | N-Myc | - | Nkx-2 |
| **VII** | 8.5 | - | GATA-2 | v-Myb | N-Myc | - | - |
| **VIII** | 3 | - | GATA-2 | v-Myb | - | - | - |
| **IX** | 6 | CdxA | - | v-Myb | N-Myc | - | Nkx-2 |
| **X** | 3 | CdxA | - | v-Myb | N-Myc | - | - |
| **XI** | 1 | CdxA | - | v-Myb | - | - | - |
| **XII** | 1 | CdxA | - | v-Myb | N-Myc | - | - |
| **XIII** | 17 | - | GATA-2 | Tst-1, CdxA | N-Myc | Oct-1, C/EBP | Nkx-2 |
| **XIV** | 1 | - | GATA-2 | Tst-1, CdxA | N-Myc | Oct-1, C/EBP | Nkx-2 |
| **XV** | 1 | - | - | Tst-1, CdxA | N-Myc | Oct-1, C/EBP | Nkx-2 |
| **XVI** | 1 | - | - | v-Myb | N-Myc | - | Nkx-2 |
| **XVII** | 1 | - | GATA-2 | v-Myb | N-Myc | - | - |
| **XVIII** | 1.5 | - | GATA-2 | v-Myb | N-Myc | - | Nkx-2 |
| **XIX** | 2 | - | - | v-Myb | N-Myc | - | - |
| **XX** | 1.5 | - | - | v-Myb | - | - | - |
| **Others** | 4.5 |  |  |  |  |  |  |
| **Undetermined** | 8.5 |  |  |  |  |  |  |
