# Supplementary material for: Toll-Like Receptor 4 Promoter Polymorphisms: Common TLR4 Variants May Protect against Severe Urinary Tract Infection
Source: PLoS One. 2010 May 20;5(5):e10734. doi: 10.1371/journal.pone.0010734 (PMC2873976; doi:10.1371/journal.pone.0010734)
Supplement: Table S8 — Genotype Pattern (GP) frequency in adult UTI prone patients and UTI free controls. (0.06 MB DOC) [file pone.0010734.s009.doc]

**Table S8.** Genotype Pattern (GP) frequency in adult UTI prone patients and UTI free controls

|  | **Adult patients and controls** | | | | |
| --- | --- | --- | --- | --- | --- |
|  | **Secondary ABU** | **APN** | **APN** |  | **Controls** |
| GP | 3 (15) | 0.7734 | 9 (21) | 0.6966 | 50 (25) |
| IV | 0 (0) | 1.0000 | 2 (5) | 0.6304 | 6 (3) |
| V | 3 (15) | 0.1908 | 6 (14) | 0.1278 | 14 (7) |
| VI | 0 (0) | 0.3761 | 4 (10) | 0.7677 | 17 (9) |
| VII | 5 (25) | 0.0113 | 7 (17) | 0.0285 | 12 (6) |
| IX | 1 (5) | 0.4918 | 4 (10) | 0.0748 | 6 (3) |
| X | 2 (10) | 0.5414 | 3 (7) | 0.1550 | 34 (17) |
| XIII | 1 (5) | 0.2497 | 0 (0) | 1.0000 | 2 (1) |
| XVI | 0 (0) | 1.0000 | 1 (2) | 0.5359 | 3 (2) |
| XX | 5 (25) | 0.5611 | 6 (14) | 0.5177 | 39 (20) |
| Others | 0 (0) |  | 0 (0) |  | 17 (9) |
| Undetermined | 3 (15) | 0.7734 | 9 (21) | 0.6966 | 50 (25) |

2x2 contingency table with Fisher's Exact Test; comparison between patient groups and controls
